# Tissue factor/factor VIIa signalling promotes cytokine-induced beta cell death and impairs glucose-stimulated insulin secretion from human pancreatic islets

**DOI:** 10.1007/s00125-015-3729-y

**Published:** 2015-08-14

**Authors:** Desirée Edén, Agneta Siegbahn, Dariush Mokhtari

**Affiliations:** Department of Medical Sciences, Clinical Chemistry, Science for Life Laboratory, University Hospital, Uppsala University, Entr. 61 3rd floor, S-751 85 Uppsala, Sweden

**Keywords:** Beta cells, Cytokines, Diabetes, FVIIa, Glucose-stimulated insulin secretion, JNK, Pancreatic islets, Tissue factor

## Abstract

**Aims/hypothesis:**

Patients diagnosed with type 1 or type 2 diabetes have elevated levels of coagulation factor VIIa (FVIIa) and its receptor tissue factor (TF) in their bloodstream. This may affect the fate of the beta cells. We aimed to study the effects of TF/FVIIa signalling on cytokine-induced beta cell death and islet function in vitro.

**Methods:**

Human pancreatic islets and MIN-6 beta cells were used to study TF mRNA and protein expression using real-time PCR, immunoblotting and flow cytometry. The effects of TF/FVIIa on cytokine-induced beta cell death were studied in MIN-6 cells and human pancreatic islets using cell-death ELISA and propidium iodide and cleaved caspase-3 staining. Effects of TF/FVIIa on the phosphorylation of p38, extracellular signal-regulated kinase and c-Jun N-terminal kinase (JNK) were investigated by immunoblotting. Glucose-stimulated insulin secretion (GSIS) from human islets was measured with an insulin ELISA.

**Results:**

A combination of the cytokines IL-1β, TNF-α and IFN-γ induced TF expression in human pancreatic islets and in beta cells. TF/FVIIa did not affect basal beta cell death but, independently of downstream coagulation activity, augmented beta cell death in response to cytokines. The effect of TF/FVIIa on cytokine-induced beta cell death was found to be dependent on the stress kinase JNK, since FVIIa addition potentiated cytokine-induced JNK activation and JNK inhibition abolished the effect of TF/FVIIa on cytokine-induced beta cell death. Moreover, TF/FVIIa signalling resulted in inhibition of GSIS from human pancreatic islets.

**Conclusions/interpretation:**

These results indicate that TF/FVIIa signalling has a negative effect on beta cell function and promotes beta cell death in response to cytokines.

## Introduction

Type 1 and type 2 diabetes are characterised by progressive loss and failure of the insulin-producing beta cells. In type 1 diabetes, the loss of beta cells is thought to result from destruction of the beta cells located within the pancreatic islets of Langerhans [1]. Upon activation, immune cells release pro-inflammatory cytokines, such as IL-1β, TNF-α and IFN-γ, believed to facilitate the dysfunction and death of beta cells [1]. Beta cell death also contributes to type 2 diabetes, although the pathogenesis is more variable and involves beta cell death or failure and peripheral insulin resistance [2, 3].

Tissue factor (TF) is a transmembrane protein considered to be the main regulator of haemostasis and thrombosis. TF has mostly been studied for its role in blood clotting where, after vascular injury or certain disease states, it binds coagulation factor VIIa (FVIIa) and initiates the coagulation cascade [4]. Beyond promoting blood clotting, TF/FVIIa can, via intracellular signalling, contribute to processes such as tumour growth and inflammation [4–6]. Islets and beta cells express TF under normal conditions [7, 8] and islet TF has been studied in the field of islet transplantation, wherein TF coagulant activity promotes clot formation, leading to islet cell death after transplantation [8, 9]. Since diabetic patients show increased plasma levels of TF and FVIIa, it is likely that the beta cells of these individuals are subjected to TF/FVIIa signalling [10–15]. However, the outcome of TF/FVIIa signalling on the viability and function of beta cells has not been studied before. In the present study, we provide evidence that TF/FVIIa signalling, independently from coagulation, impairs pancreatic islet function and augments beta cell death in response to cytokines.

## Methods

### Reagents

CMRL 1066, DMEM, FCS, Trizol, Lipofectamine RNAiMAX, Taqman probes and oligoDT primers were from Life Technologies, Carlsbad, CA, USA. Hirudin, coagulation factor Xa (Fxa) and Hoechst stain (33258) were from Sigma-Aldrich, St Louis, MO, USA. Cytokines and IL-1 receptor antagonist (IL-1 Ra) were from R&D systems, Minneapolis, MN, USA. Cell Death Detection ELISA^PLUS^ was from Roche, Basel, Switzerland. Human and murine FVIIa were kind gifts from L. C. Petersen (Novo Nordisk, Bagsvaerd, Denmark). Small interfering RNAs (siRNAs) were from Thermo scientific, Waltham, MA, USA. Insulin ELISA was from Mercodia, Uppsala, Sweden. SB203580, PD98059 and SP600125 were from Tocris Bioscience, Bristol, UK. The following antibodies (Ab) were from Cell Signaling Technology, Danvers, MA, USA: rabbit phosphorylated extracellular signal-regulated kinase (phospho-ERK) (no. 4370), rabbit phosphorylated c-Jun N-terminal kinase (phospho-JNK) (no. 4668), rabbit phospho-p38 (no. 4631), mouse ERK (no. 9107), mouse JNK (no. 3708), mouse p38 (no. 9217), rabbit glyceraldehyde-3-phosphate dehydrogenase (GAPDH) (no. 2118) and rabbit cleaved caspase-3 (no. 9661). Rabbit TF Ab (Ref. 4515) was from Sekisui Diagnostics, Lexington, MA, USA. Guinea pig insulin Ab (Ab7842) was from Abcam, Cambridge, UK. Mouse TF Ab (clone TF9-10H10) was from AbD Serotec, Kiddlington, UK. Goat TF Ab (no. AF3178) and goat IgG (no. AB-108-C) were from R&D systems. Rabbit TF Ab (no. N1C3) was from GeneTex, Irvine, CA, USA.

### Islets and cells

Human islets were provided by the Uppsala facility for the isolation of human islets. After isolation, the islets were cultured free-floating in Sterilin dishes in CMRL 1066 medium supplemented with 10% FCS, l-glutamine, benzylpenicillin and streptomycin. Glucose responsive MIN-6 cells were a kind gift from O. Idevall at the Department of Medical Cell Biology, Uppsala University, Sweden. Before use, cells tested negative for mycoplasma contamination using MycoSEQ Mycoplasma Detection Kit (Life Technologies). MIN-6 cells (passages 20–30) were grown in DMEM medium supplemented as above with the addition of 70 μmol/l β-mercaptoethanol. Islets and cells were cultured in a humidified chamber at 37°C and 5% CO_2_.

### mRNA analysis

Real-time quantitative PCR analyses of *TF* (also known as *F3*) and the gene encoding ribosomal protein, large, P0 (*RPLP0*) were performed on cDNA from oligoDT converted total RNA extracted by Trizol according to standard protocols.

### FVIIa and cytokines

FVIIa activity was validated by measuring TF/FVIIa specific upregulation of *IL8* mRNA in MDA-MB-231 cells [16]. A concentration of 10 or 50 nmol/l was used to achieve maximal activation of TF/FVIIa signalling [16, 17]. Cells were stimulated with IL-1β (50 U/ml), TNF-α (1,000 U/ml) and IFN-γ (1,000 U/ml) individually or combined into a cytokine mixture. FVIIa was present during cytokine exposure and culture media was changed and fresh cytokines and FVIIa added every 24 h.

### TF cell surface expression

Cells were incubated with 10 μg/ml mouse TF Ab (no. AF3178) or normal 10 μg goat IgG control (no. AB-108-C) for 1 h at room temperature and then washed in PBS containing 1% (wt/vol.) BSA. The cells were then incubated with Alexa Fluor 488 donkey anti-goat secondary antibody (1/800 dilution) for 30 min at room temperature before being analysed using a flow cytometer.

### Transfections

Cells were transfected using Lipofectamine RNAiMAX with 10 nmol/l siRNA against mouse *Tf* or scrambled siRNA according to the instructions of the supplier. The cells were re-transfected after 24 h.

### Determination of beta cell death

Cell death in MIN-6 cells was evaluated using a Cell Death ELISA^PLUS^, detecting enrichment of cytoplasmic nucleosomes or by propidium iodide (PI) staining. Cell death in human islets was studied by insulin and cleaved caspase-3 staining. To study single beta cells, islets were mildly dispersed using trypsin. Single islet cells were cytospinned to microscope slides, fixed in 4% paraformaldehyde, blocked, stained using insulin (1/50 dilution) and cleaved caspase-3 (1/50 dilution) antibodies and analysed under a Zeiss Axiovert 40CFL microscope (Carl Zeiss AG, Oberkochen, Germany).

### Immunoblotting

Cells were lysed in SDS sample buffer and separated by SDS-PAGE. Proteins were transferred to Immobilon-FL PVDF membranes, blocked in Blok-FL (Merck Millipore, Billerica, MA, USA) and left overnight at 4°C in Blok-FL with antibodies diluted 1/1,000. Membranes were washed in Tris-buffered saline containing 0.01% (vol./vol.) Tween 20 and incubated with secondary antibodies (1/10,000 dilution). Immunodetection was performed in Odyssey Imaging System (LI-COR, Lincoln, NE, USA) and band intensities quantified by densitometric scanning using the Odyssey V3.0 software (LI-COR).

### Insulin secretion

Islets were incubated for 60 min in Krebs-Ringer bicarbonate HEPES buffer (KRBH) containing 1.67 mmol/l glucose followed by 60 min in KRBH containing 16.7 mmol/l glucose. Media and islets were recovered and insulin concentration was determined using a human insulin ELISA. For protocol see [18].

### Ethics statement

Studies involving human islets were approved by the local ethical board in Uppsala, Sweden.

### Statistics

Statistical significances were obtained by comparison with the corresponding control using Student’s paired *t* test and GraphPad prism software 6.0 (GraphPad software, San Diego, CA, USA)

## Results

### Cytokines induce TF expression in human islets and MIN-6 beta cells

**Fig. 1:**
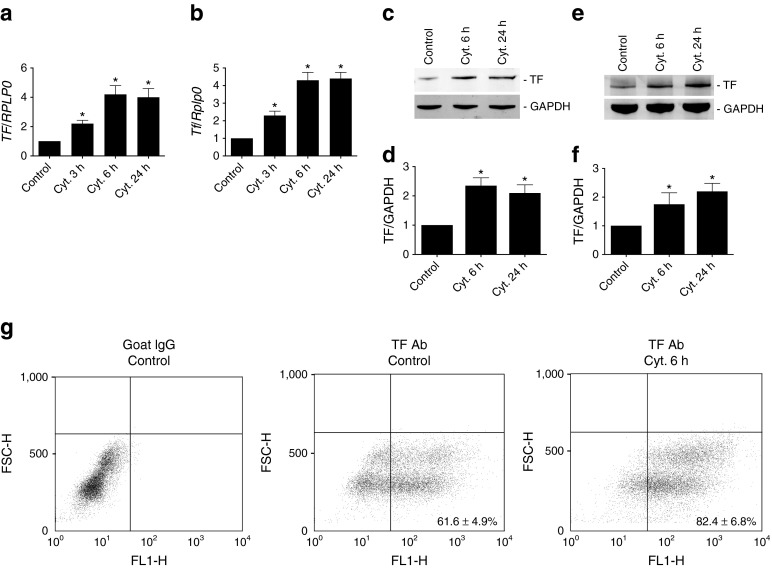
Cytokines induce TF expression in human islets and MIN-6 cells. Human islets and MIN-6 cells were left untreated (Control) or treated with a cytokine mixture of IL-1β + TNF-α + IFN-γ (Cyt.) for the indicated time periods. *TF* mRNA levels related to *RPLP0* in (**a**) human islets and (**b**) MIN-6 cells. Immunoblot and graphs showing TF/GAPDH ratio using TF and GAPDH antibodies on (**c**, **d**) human islets and (**e**, **f**) MIN-6 cells. (**g**) MIN-6 cells were left untreated (Control) or were treated with cytokine mixture (Cyt.) for 6 h. Cells were incubated with mouse TF antibody (Ab) and TF cell surface expression was analysed using flow cytometry detecting forward scatter height (FSC-H) and fluorescence in fluorescence channel 1 height (FL1-H). Normal goat IgG was used as isotype control. Results are means ± SEM from four (**a**–**f**) or three (**g**) independent experiments. *p < 0.05 vs control, using paired Student’s *t* test

We first studied the influence of cytokines on TF expression in islets and beta cells. In human islets and MIN-6 cells, a significant increase in *TF* mRNA levels (≈ twofold) was observed following 3 h of exposure to a cytokine mixture (IL-1β + TNF-α + IFN-γ) when compared with control (Fig. 1a, b). A further increase (≈ fourfold) was seen after exposure to cytokines for 6 and 24 h (Fig. 1a, b). This was paralleled by an induction in TF protein levels in human islets and MIN-6 cells following 6 and 24 h of cytokine exposure (Fig. 1c–f). A large pool (61.6 ± 4.9%) of untreated MIN-6 cells stained positive for TF cell surface expression (Fig. 1g). Cytokine treatment for 6 h increased the number of TF-positive cells (82.4 ± 6.8%) when compared with untreated cells (Fig. 1g), indicating that TF total and cell surface expression in beta cells are upregulated in the presence of cytokines.

### TF/FVIIa signalling augments cytokine-induced beta cell death independently of downstream coagulation

**Fig. 2:**
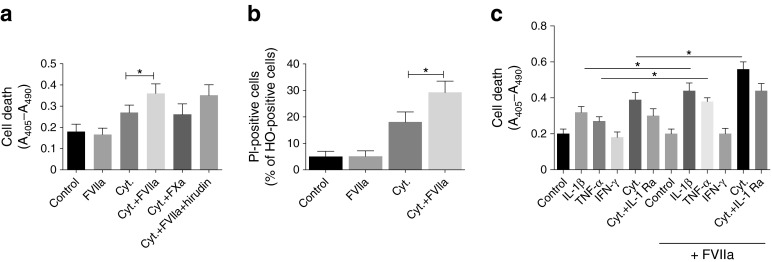
TF/FVIIa signalling augments cytokine-induced beta cell death independently of downstream coagulation. MIN-6 cells were pre-treated with FVIIa (10 nmol/l), FXa (100 nmol/l) or FVIIa+hirudin (10 U/ml) for 6 h, followed by treatment with a cytokine mixture of IL-1β, TNF-α and IFN-γ (Cyt.) for 72 h. (**a**) Cell death was quantified using a cell-death ELISA by measuring absorbance (A_405_–A_490_). (**b**) Cells were stained with PI and Hoechst 33 258 (HO), ~2,000 cells/group were counted and the number of PI-positive cells was determined by fluorescence microscopy. (**c**) MIN-6 cells were first pre-treated with FVIIa (10 nmol/l) for 6 h. Indicated groups were then treated with IL-1 Ra (500 ng/ml) for 30 min followed by treatment with individual cytokines or with cytokine mixture for 72 h. Cell death was quantified using a cell-death ELISA by measuring absorbance (A_405_–A_490_). Results are means ± SEM from four experiments. *p < 0.05 for indicated comparisons, using paired Student’s *t* test

We next studied the effects of FVIIa on cytokine-induced beta cell death, evaluated by a cell-death ELISA and PI staining. Treatment of MIN-6 cells with cytokine mixture led to an increase in cell death (Fig. 2). FVIIa treatment alone had no significant effect on cell death (Fig. 2). Conversely, FVIIa pre-treatment significantly potentiated cytokine-induced cell death (Fig. 2). Addition of FXa had no effect on cytokine-induced beta cell death and the thrombin inhibitor hirudin failed to prevent the potentiation of cytokine-induced beta cell death induced by FVIIa (Fig. 2a).

We also studied the effects of the individual cytokines IL-1β, TNF-α and IFN-γ on beta cell death and on TF/FVIIa signalling. IL-1β treatment alone led to a significant increase in cell death and TNF-α, albeit not reaching statistical significance, increased cell death (Fig. 2c). IFN-γ treatment alone did not affect cell viability (Fig. 2c). The cytokine mixture further potentiated beta cell death when compared with IL-1β or TNF-α treatment alone. FVIIa pre-treatment significantly augmented beta cell death in response to IL-1β or TNF-α alone or to cytokine mixture but failed to promote beta cell death in response to IFN-γ (Fig. 2c). Moreover, IL-1 Ra partially inhibited cell death induced by the cytokine mixture in both untreated and FVIIa pre-treated cells (Fig. 2c).

**Fig. 3:**
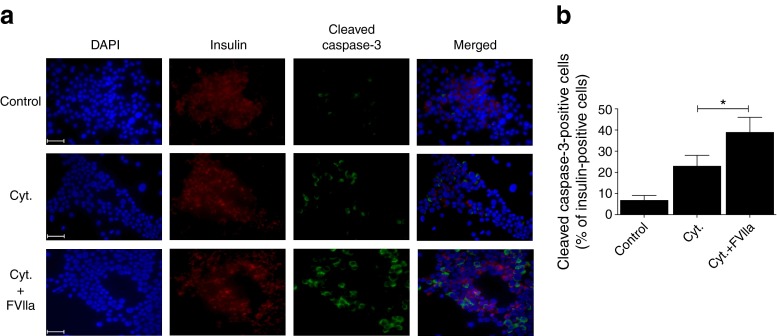
FVIIa promotes cytokine-induced caspase-3 cleavage in primary human beta cells. (**a**) Untreated or FVIIa pre-treated (10 nmol/l, 6 h) human islets were stimulated with a cytokine mixture of IL-1β, TNF-α and IFN-γ (Cyt.) for 72 h, then mildly dispersed using trypsin to generate single islet cells. Cells were stained with DAPI (blue), insulin antibody (red) and cleaved caspase-3 antibody (green) and visualised by fluorescence microscopy. Islets from five different donors were used and images show results from one representative donor. Scale bar, 50 μm. (**b**) Cells (~2,000/group) were counted and the number of cells double-positive for insulin and cleaved caspase-3 are depicted in the graph. Results are means ± SEM from five experiments. *p < 0.05 for indicated comparison, using paired Student’s *t* test

Finally, we investigated the effects of FVIIa in primary human islet cells. Untreated islets or islets treated with cytokine mixture for 72 h were dispersed to generate single islet cells and then co-stained using insulin and cleaved caspase-3 antibodies. The number of insulin-positive cells varied between 30% and 80% using five different donor preparations (Fig. 3). The cytokine mixture induced caspase-3 cleavage in beta cells when compared with control cells and caspase-3 cleavage was further increased by FVIIa pre-treatment (Fig. 3b).

### TF downregulation blocks the effect of FVIIa on cytokine-induced beta cell death

**Fig. 4:**
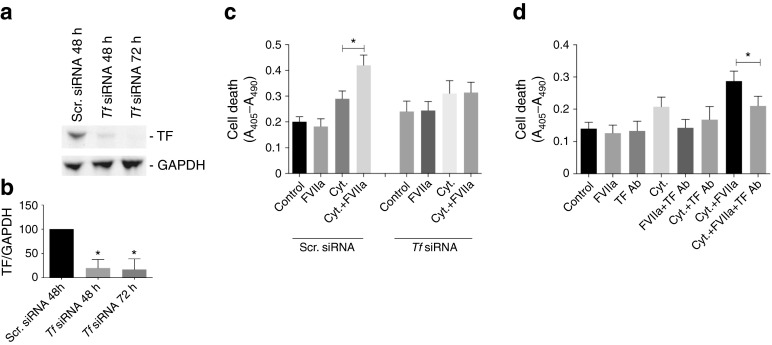
TF downregulation blocks the effect of FVIIa on cytokine-induced beta cell death. (**a**, **b**) MIN-6 cells were transfected with scrambled (Scr.) or *Tf* siRNA. At 48 or 72 h post transfection, cells were subjected to immunoblotting using TF and GAPDH antibodies. (**c**) At 48 h after siRNA transfection, MIN-6 cells were pre-treated with FVIIa (10 nmol/l) for 6 h followed by treatment with a cytokine mixture of IL-1β, TNF-α and IFN-γ (Cyt.) for 72 h. Cell death was quantified using a cell-death ELISA measuring absorbance (A_405_–A_490_). (**d**) MIN-6 cells were cultured in the absence or presence of TF antibody (Ab) 4515 (100 μg/ml, 1 h). Cells were left untreated or were treated with FVIIa (10 nmol/l) for 6 h and then cytokine mixture for 72 h. Cell death was quantified using a cell-death ELISA measuring absorbance (A_405_–A_490_). Results are means ± SEM from three (**a, b**) or four (**c**, **d**) individual experiments. *p < 0.05 for indicated comparisons, using paired Student’s *t* test

To determine whether the effects of FVIIa are dependent on TF/FVIIa, we downregulated TF protein levels (Fig. 4a, b). FVIIa potentiated cell death induced by the cytokine mixture in MIN-6 cells transfected with scrambled siRNA, when analysed using a cell-death ELISA (Fig. 4c). *Tf* siRNA prevented FVIIa-induced potentiation of cytokine-induced cell death but did not restore cell death to control levels (Fig. 4c). In addition, we used a TF antibody to block TF/FVIIa. We observed a decrease in cytokine-induced cell death after administration of TF antibody alone (Fig. 4d). Moreover, TF antibody counteracted the potentiation of cytokines induced by FVIIa but failed to restore cell death to control levels (Fig. 4d).

### TF/FVIIa signalling promotes cytokine-induced beta cell death via activation of the stress kinase JNK

**Fig. 5:**
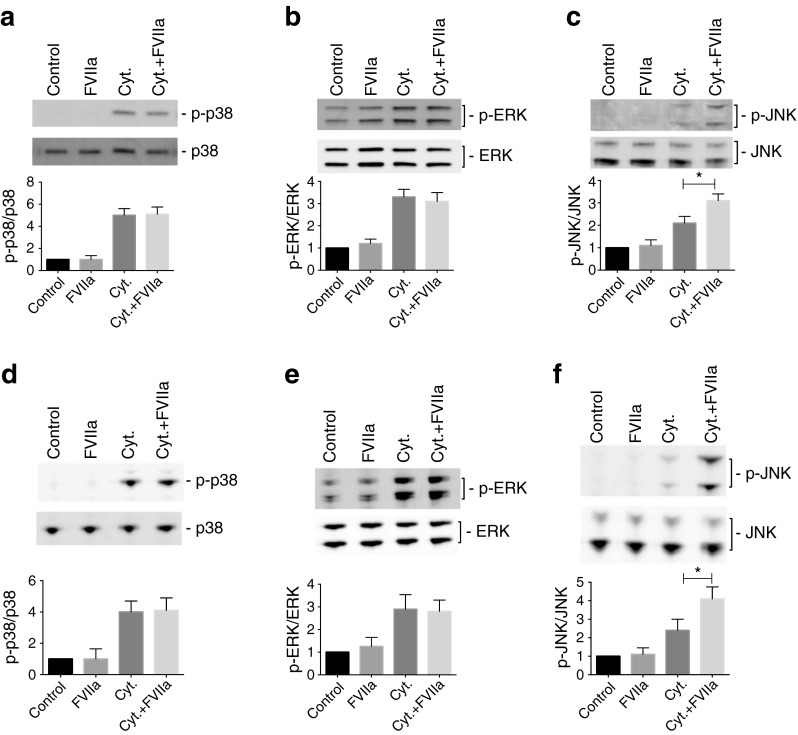
TF/FVIIa signalling promotes cytokine-induced activation of the stress kinase JNK. Human islets (**a**–**c**) and MIN-6 cells (**d**–**f**) were either left untreated (Control) or pre-treated with FVIIa (50 nmol/l for human islets and 10 nmol/l for MIN-6 cells) for 30 min. Some groups were treated with a cytokine mixture of IL-1β, TNF-α and IFN-γ (Cyt.) for 15 min. Islets and cells were analysed by immunoblotting using antibodies against total and phosphorylated forms of ERK, p38 and JNK. The fold increase vs control was determined by calculating the ratio between phosphorylated and total band intensities. The two forms of JNK and ERK were quantified as one band. When phospho-p38 and phospho-JNK bands were undetectable in control groups, the background intensity was used for quantification. Results are means ± SEM from four independent experiments. *p < 0.05 for the indicated comparisons, using paired Student’s *t* test

**Fig. 6:**
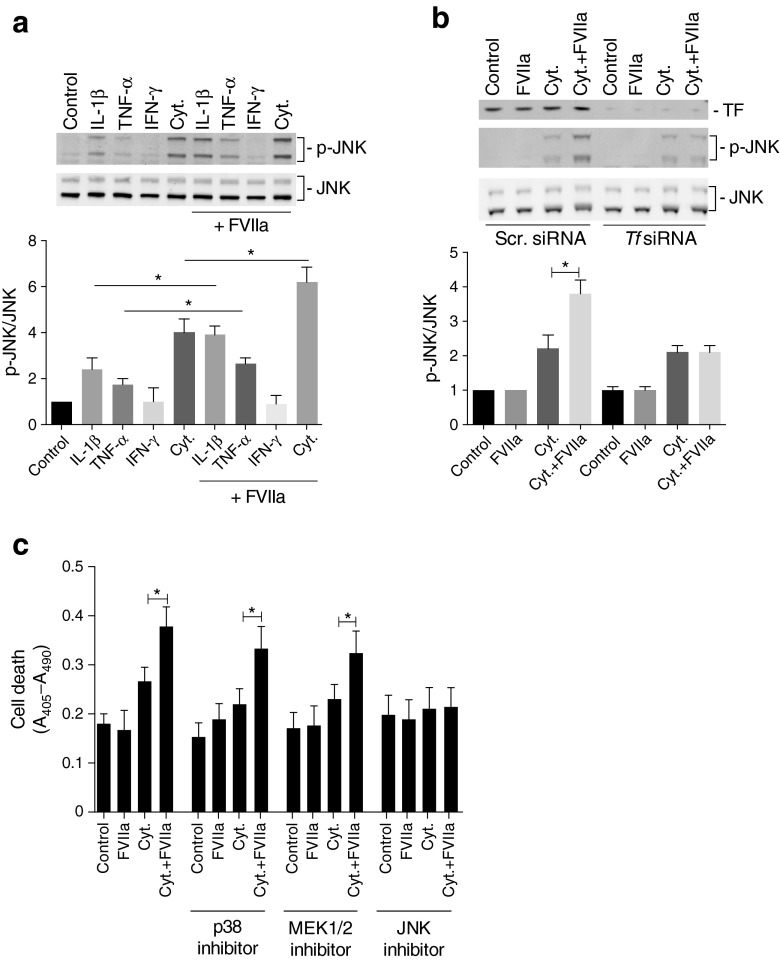
TF/FVIIa-mediated JNK activation promotes cytokine-induced beta cell death. (**a**) MIN-6 cells were pre-treated with FVIIa (10 nmol/l) for 6 h followed by treatment with individual cytokines or a cytokine mixture of IL-1β, TNF-α and IFN-γ (Cyt.) for 15 min. Cells were then lysed and subjected to immunoblotting using phospho- and total JNK antibodies. (**b**) MIN-6 cells were transfected with scrambled (Scr.) siRNA or *Tf* siRNA. At 72 h post transfection cells were stimulated with cytokine mixture for 15 min, lysed and subjected to immunoblotting using TF and JNK antibodies. (**c**) MIN-6 cells were pre-treated with FVIIa (10 nmol/l) for 6 h followed by treatment with cytokine mixture for 72 h. Thirty minutes before the addition of FVIIa, p38 inhibitor SB203580 (10 μmol/l), MEK1/2 inhibitor PD98059 (10 μmol/l) and JNK inhibitor SP600125 (10 μmol/l) were added to the indicated groups. Cell death was quantified using a cell-death ELISA measuring absorbance (A_405_–A_490_). Results are means ± SD from three (**a**, **b**) or four (**c**) individual experiments. *p < 0.05 for indicated comparisons, using paired Student’s *t* test

To identify the mechanisms behind the effects of TF/FVIIa on cytokine-induced beta cell death, we studied the consequences of cytokine and FVIIa treatment on mitogen-activated protein kinase (MAPK) activation. Treatment with cytokines resulted in increased phosphorylation of p38, ERK and JNK in islets and MIN-6 beta cells (Fig. 5). FVIIa did not alter ERK or p38 phosphorylation in response to cytokines, but augmented cytokine-induced JNK phosphorylation (Fig. 5), an event that was abolished by *Tf* siRNA (Fig. 6b). FVIIa did not affect phosphorylation of p38, ERK or JNK at basal conditions (Fig. 5).

We also studied the contribution of individual cytokines to JNK activation. IL-1β and, to a lesser extent, TNF-α individually promoted JNK phosphorylation in MIN-6 cells, whereas IFN-γ did not (Fig. 6a). The cytokine mixture further increased JNK phosphorylation when compared with IL-1β or TNF-α alone (Fig. 6a) and FVIIa pre-treatment augmented JNK activation in response to IL-1β, TNF-α and the cytokine mixture (Fig. 6a).

To determine the functional importance of TF/FVIIa-mediated JNK activation in response to cytokines, we next studied how MAPK activity affected cytokine-induced beta cell death. MIN-6 beta cells were cultured in the absence or presence of inhibitors of p38, MEK1/2 (ERK) or JNK prior to addition of FVIIa and cytokine mixture. Inhibition of p38, ERK or JNK alone or in combination with FVIIa did not affect basal beta cell death whereas all inhibitors mitigated cytokine-induced beta cell death (Fig. 6c). The effect of FVIIa on cytokine-induced beta cell death was not changed by inhibition of p38 or ERK but was abolished in the presence of JNK inhibition, suggesting that TF/FVIIa mediates beta cell death in response to cytokines by mechanisms involving activation of the stress kinase JNK (Fig. 6c).

### TF/FVIIa signalling impairs glucose-stimulated insulin secretion from human pancreatic islets

**Fig. 7:**
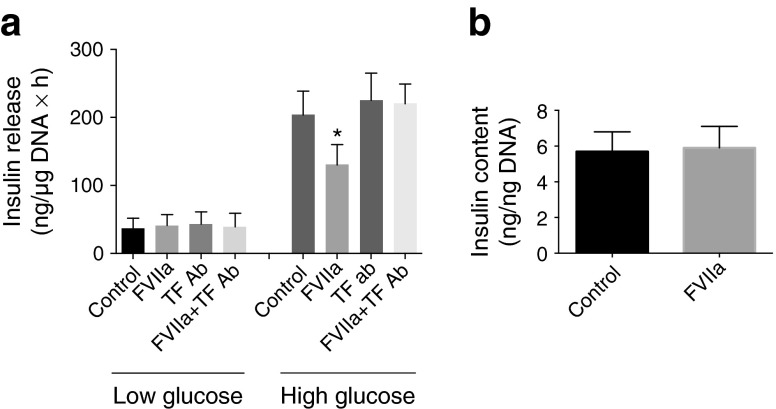
TF/FVIIa impairs GSIS from human pancreatic islets. (**a**) Human islets in groups of 10 in triplicates were either left untreated or treated with FVIIa (50 μmol/l, 3 h). To some groups TF antibody 10H10 (TF Ab) (50 μg/ml) was added 30 min before the FVIIa. Insulin release from islets to the culture media in response to low glucose (1.67 mmol/l, 1 h) and high glucose (16.7 mmol/l, 1 h) was quantified using ELISA. Values are normalised to the amount of DNA/sample. Results are mean values from triplicates of islets from three different donors. *p < 0.05 vs high-glucose control, using paired Student’s *t* test. (**b**) Islets from (**a**) were recovered and insulin content was determined using ELISA

To find out how TF/FVIIa signalling influences the function of beta cells, we measured glucose-stimulated insulin secretion (GSIS) from human islets using ELISA. When normalised to islet DNA content, islets pre-treated with FVIIa secreted a similar amount of insulin to that secreted by control islets when cultured in a low-glucose concentration (Fig. 7a). However, when cultured in an increased concentration of glucose, there was an impaired insulin secretion in islets treated with FVIIa when compared with control islets (Fig. 7a). Furthermore, the inhibition of GSIS by FVIIa was counteracted by TF antibody 10H10. Islet insulin content was similar in FVIIa-treated and control islets (Fig. 7b).

## Discussion

We herein report that cytokines promote TF expression in islets and beta cells and that TF/FVIIa signalling impairs islet function and augments beta cell death in response to cytokines. Our findings provide evidence for and mechanistic insight into how TF/FVIIa signalling could contribute to beta cell destruction and diabetes.

Inflammation is believed to be a key feature in the pathogenesis of diabetes and cytokines are known to contribute to the failure and death of beta cells [2]. Since TF/FVIIa, via coagulation or intracellular signalling can result in inflammatory responses [4, 19], we investigated in vitro its role in cytokine-induced beta cell death, in order to understand the molecular mechanism involved in its action. This is relevant since several clinical observations support a role for TF/FVII in diabetes. Plasma levels of TF antigen, TF activity, TF-containing microparticles and FVIIa are elevated in patients with type 1 and type 2 diabetes when compared with control individuals [10–15] and glucose-lowering drugs such as metformin and exenatide downregulate TF expression [20, 21].

Our data suggest that FVIIa promotes cytokine-induced beta cell death by binding to TF. TF downregulation and a TF antibody abolished the effect of FVIIa on cytokine-induced beta cell death and it is therefore unlikely that the effect is mediated through a TF-independent mechanism. We noted that the TF antibody provided protection against cytokine-induced beta cell death even in the absence of FVIIa stimulation. The reason for this remains unclear but it has been reported that cancer cells can express FVII/FVIIa [22]. One possible explanation could therefore be that MIN-6 cells produce FVII/FVIIa. TF/FVIIa is known to mediate cellular signalling that is uncoupled to FXa or thrombin generation [23]. Consistent with this, the effect of TF/FVIIa on cytokine-induced beta cell death was found to be independent of downstream coagulation, since addition of FXa did not affect beta cell death in response to cytokines and the thrombin inhibitor hirudin failed to abrogate the effect of FVIIa.

It appears that TF/FVIIa signalling affects both the IL-1β and the TNF-α pathway. FVIIa stimulation alone did not affect basal cell death whereas it augmented beta cell death when combined with IL-1β or TNF-α individually or with cytokine mixture. Interestingly, TF has been associated with anti-apoptotic effects in several non-islet cells [23]. With regards to this, we did note that cells treated with *Tf*siRNA, under basal conditions, showed a small increase in cell death compared with control cells. It might therefore be that TF promotes survival of unstressed beta cells but that it mediates cell death when the beta cells are challenged with inflammatory stress and FVIIa binding.

Our results support the notion that cytokines have cooperative effects on beta cells. We found that treatment with cytokine mixture further increased beta cell death when compared with the effects of IL-1β or TNF-α alone. Moreover, IL-1 Ra only partially blocked cell death induced by the cytokine mixture. In our experiments, IFN-γ alone did not induce beta cell death. This is in agreement with data showing that IFN-γ by itself is not detrimental to beta cells but when combined with IL-1β or TNF-α it promotes beta cell death [24].

We propose JNK to be the main mediator of the TF/FVIIa signalling effect. It is known that p38 and JNK mediate beta cell death in vitro in response to cytokines and that this event can be blocked by inhibition of p38 or JNK [25–28]. Treatment with the cytokine mixture led to phosphorylation of p38, ERK and JNK. FVIIa pre-treatment did not affect cytokine-induced ERK and p38 phosphorylation but augmented cytokine-induced JNK phosphorylation. Also, IL-1β and TNF-α individually promoted JNK phosphorylation in MIN-6 cells and maximal JNK activity was observed in cells treated with cytokine mixture, indicating a synergistic effect of cytokines on beta cells. Moreover, JNK inhibition abolished the effect of FVIIa on cytokine-induced beta cell death, indicating that JNK activation is necessary for the effects of TF/FVIIa on cytokine-induced beta cell death. JNK activation also contributes to insulin resistance and JNK activity in macrophages is necessary for the development of diet-induced insulin resistance [29–32]. Intriguingly, TF/FVIIa signalling promotes high-fat diet-induced obesity, adipose tissue inflammation and insulin resistance in mice [6]. TF/FVIIa-induced JNK activity could therefore not only promote beta cell death in response to cytokines but also contribute to peripheral insulin resistance.

We also report that TF/FVIIa signalling impairs beta cell function. Since FVIIa-treated islets and control islets contained a similar amount of insulin, the effect of TF/FVIIa on GSIS cannot be accounted for by differences in insulin storage. Instead, our results favour the view that TF/FVIIa signalling somehow perturbs the stimulus–secretion coupling process. In these experiments, the TF antibody 10H10 blocked the detrimental effects of FVIIa on GSIS. Given that the 10H10 antibody mainly targets TF/FVIIa signalling by inhibition of protease-activated receptor 2 (PAR-2) cleavage, the effect of TF/FVIIa on GSIS is likely to be mediated via a PAR-2-dependent pathway [33]. Consistent with this view, Regard et al [34] and Lim et al [35] showed that PAR-2 activation leads to inhibition of GSIS from MIN-6 beta cells.

Beta cells in vivo are exposed to a variety of cytokines. We demonstrate that TF expression in islets and beta cells is induced by a combination of the cytokines IL-1β, TNF-α and IFN-γ. This is in line with studies demonstrating that cytokines promote TF expression in several non-islet cell types [36–38]. The induction of TF is probably mediated by nuclear factor κ-light-chain enhancer of activated B cells (NF-κβ). IL-1β and TNF-α promote NF-κβ activation in beta cells and TF expression can be rapidly induced by activation of NF-κβ in other cell types [39–41].

The mechanisms by which TF/FVIIa promotes JNK phosphorylation in response to cytokines in beta cells remain unknown but may involve activation of PAR-2. PAR-2 signalling mediates inflammatory responses in various cell types and can result in JNK activation in keratinocytes [42]. JNK activity is regulated by upstream MAP3Ks and different MAP3Ks, such as apoptosis signal-regulating kinase 1 and MAPK kinase kinase 1, promote JNK activation in a cell type- and stimulus-specific manner [41]. Future studies will hopefully delineate the mechanism by which TF/FVIIa promotes JNK activity in beta cells.

To conclude, we provide evidence that TF expression in islets and beta cells is induced by cytokines and propose that TF/FVIIa signalling promotes cytokine-induced beta cell death and inhibits islet function. Inhibiting TF/FVIIa signalling (e.g. by using a TF blocking antibody that has minimal effect on coagulation) could be a novel way to promote beta cell survival and function.

## Abbreviations

ERK: Extracellular signal-regulated kinase
FVIIa: Coagulation factor VIIa
FXa: Coagulation factor Xa
GAPDH: Glyceraldehyde-3-phosphate dehydrogenase
GSIS: Glucose-stimulated insulin secretion
IL-1 Ra: IL-1 receptor antagonist
JNK: c-Jun N-terminal kinase
KRBH: Krebs-Ringer bicarbonate HEPES buffer
LPS: Lipopolysaccharide
MAPK: Mitogen-activated protein kinase
NF-κβ: Nuclear factor κ-light-chain-enhancer of activated B cells
PAR-2: Protease-activated receptor 2
PI: Propidium iodide
RPLP0: Ribosomal protein large, P0
siRNA: Small interfering RNA
TF: Tissue factor
